# Program Completion of a Web-Based Tailored Lifestyle Intervention for Adults: Differences between a Sequential and a Simultaneous Approach

**DOI:** 10.2196/jmir.1968

**Published:** 2012-03-08

**Authors:** Daniela N Schulz, Francine Schneider, Hein de Vries, Liesbeth ADM van Osch, Peter WM van Nierop, Stef PJ Kremers

**Affiliations:** ^1^CAPHRI School for Public Health and Primary CareDepartment of Health PromotionMaastricht UniversityMaastrichtNetherlands; ^2^Municipal Health ServicesDepartment of Health PromotionHelmondNetherlands; ^3^Nutrition and Toxicology Research Institute Maastricht (NUTRIM)Department of Health PromotionMaastricht UniversityMaastrichtNetherlands

**Keywords:** Internet, dropout, computer tailoring, multiple health behavior change intervention, sequential, simultaneous, lifestyle

## Abstract

**Background:**

Unhealthy lifestyle behaviors often co-occur and are related to chronic diseases. One effective method to change multiple lifestyle behaviors is web-based computer tailoring. Dropout from Internet interventions, however, is rather high, and it is challenging to retain participants in web-based tailored programs, especially programs targeting multiple behaviors. To date, it is unknown how much information people can handle in one session while taking part in a multiple behavior change intervention, which could be presented either sequentially (one behavior at a time) or simultaneously (all behaviors at once).

**Objectives:**

The first objective was to compare dropout rates of 2 computer-tailored interventions: a sequential and a simultaneous strategy. The second objective was to assess which personal characteristics are associated with completion rates of the 2 interventions.

**Methods:**

Using an RCT design, demographics, health status, physical activity, vegetable consumption, fruit consumption, alcohol intake, and smoking were self-assessed through web-based questionnaires among 3473 adults, recruited through Regional Health Authorities in the Netherlands in the autumn of 2009. First, a health risk appraisal was offered, indicating whether respondents were meeting the 5 national health guidelines. Second, psychosocial determinants of the lifestyle behaviors were assessed and personal advice was provided, about one or more lifestyle behaviors.

**Results:**

Our findings indicate a high non-completion rate for both types of intervention (71.0%; n = 2167), with more incompletes in the simultaneous intervention (77.1%; n = 1169) than in the sequential intervention (65.0%; n = 998). In both conditions, discontinuation was predicted by a lower age (sequential condition: OR = 1.04; *P* < .001; CI = 1.02-1.05; simultaneous condition: OR = 1.04; *P* < .001; CI = 1.02-1.05) and an unhealthy lifestyle (sequential condition: OR = 0.86; *P* = .01; CI = 0.76-0.97; simultaneous condition: OR = 0.49; *P* < .001; CI = 0.42-0.58). In the sequential intervention, being male (OR = 1.27; *P* = .04; CI = 1.01-1.59) also predicted dropout. When respondents failed to adhere to at least 2 of the guidelines, those receiving the simultaneous intervention were more inclined to drop out than were those receiving the sequential intervention.

**Conclusion:**

Possible reasons for the higher dropout rate in our simultaneous intervention may be the amount of time required and information overload. Strategies to optimize program completion as well as continued use of computer-tailored interventions should be studied.

**Trial Registration:**

Dutch Trial Register NTR2168

## Introduction

Unhealthy lifestyle behaviors, such as physical inactivity, insufficient fruit and vegetable intake, high alcohol consumption, and smoking, often co-occur [1-4] and are related to chronic diseases like cardiovascular diseases and cancer [5]. In view of this co-occurrence, various studies have emphasized the need to develop interventions addressing more than one risk behavior.

One method to change lifestyle behavior is the use of computer tailoring. Positive aspects of web-based computer-tailored programs are that these can be administered in privacy and at a time that suits the respondent [6], and that they can be integrated in larger multicomponent interventions [7]. In addition to their positive effects on health behavior change [e.g. 7-10], numerous studies have shown that (web-based) tailored messages attract the public’s attention [11,12], are perceived as personally relevant [13], and are usually better read, saved, remembered, and discussed with others than non-tailored materials [7,9,14-16].

The public health impact of an intervention is defined by the program’s efficacy multiplied by its reach [7,17]. Thus, the impact is highest when an intervention is effective and has a wide reach. In view of the high rate of Internet access these days (91% in the Netherlands) [18], Internet-based programs may have the potential to reach large numbers of people. However, various studies have pointed out that the actual use of web-based interventions may be limited [19-21] and that leaving an Internet intervention prematurely is common [e.g. 22-24]. Brouwer et al. [25] reported that more than half of the visitors (Dutch adults) of their online intervention left the website within 30 seconds, while 10.5% stayed for more than 15 minutes. Hence, there is a need to identify factors associated with early discontinuation or continuation of participation in web-based programs promoting the adoption of healthy lifestyles.

Various tailoring strategies can be used to address multiple behaviors with computer-tailored interventions, such as a sequential or a simultaneous strategy. A simultaneous strategy concurrently targets multiple behaviors for intervention, while a sequential strategy targets a single behavior at a time. The few studies that have investigated the effects on behavioral change of sequential versus simultaneous strategies to provide multiple health-behavior change interventions reported inconsistent findings [26-28]. According to Vandelanotte et al. [28], the sequential strategy may be more effective than the simultaneous strategy when participants can choose the behavior on which they would prefer to receive personal feedback first, and can start with this part of the intervention, instead of the lifestyle modules being presented in a predefined order [see also 29].

In any case, behavioral change will be more likely when someone completes the whole intervention program [30], as early dropout is a hazard to the effectiveness of any intervention. Hence, when considering the use of a sequential or simultaneous approach for web-based computer-tailored interventions, it is important to study continuation rates. In both types of intervention, people receive only the modules about health behavior topics for which they are at risk in order to increase the relevance of the intervention [31]. When being at risk for at least two behaviors, people in the simultaneous intervention receive, and thus have to handle, more information at one point in time compared to a sequential intervention, in which the same amount of information is spread over time. Therefore, a simultaneous strategy, including a more complex program, is likely to require more time from the respondents and to increase the behavior change demands [31], especially when respondents fail to meet multiple guidelines. Hence, because a simultaneous approach may lead to an overload of information, such a strategy may potentially lead to higher dropout rates than a sequential strategy [31-32]. Respondents may become overwhelmed by the amount of information [33] and may perceive ego depletion, leading to a reduced capacity to change [34]. Furthermore, tailoring multiple behaviors simultaneously could fail to address any single behavior in sufficient depth [3,31,35]. On the other hand, addressing various behaviors simultaneously may optimize the occurrence of synergistic effects [36-40]. Hence, both strategies may have advantages as well as disadvantages. To our knowledge, there is no literature about the difference in completion and dropout rates between users of sequential and those of simultaneous behavior change interventions.

In addition to the problem of dropout, another important aspect is that high-risk populations (such as the less-educated and people with many unhealthy behaviors) are often insufficiently reached [e.g. 41], and it is especially those with unhealthy behavior who should engage in online health interventions and spend enough time on the website [42]. It is essential to identify the characteristics of people who complete or fail to complete online health interventions. In a study by Brouwer et al. [25], respondents who completed the program were mostly female, middle-aged (40 to 50 years), and medium-educated, and had a healthier lifestyle. This information about completers’ characteristics can be used to improve tailored programs by making them more attractive to the individual user. 

In conclusion, computer-tailored technology addressing multiple behaviors is still in its infancy [43-45]. It is unknown how much information people can and will handle in multiple behavior change interventions. In this study, we investigated the level of completion of a web-based tailored intervention addressing 5 lifestyle behaviors (physical activity, fruit consumption, vegetable consumption, alcohol intake, and smoking), and tested potential information overload by comparing dropout rates for two versions of the program, one offering a single behavior change module as part of a sequential program and one providing simultaneous tailored feedback on different behaviors. In addition, this study investigated personal predictors of dropout for the two versions of our computer-tailored program.

## Methods

### Design

In this study, which was part of a randomized controlled trial (Dutch Trial Register NTR2168), tailored information was provided to two groups, one receiving a sequential behavior tailoring condition (referred to below as sequential condition) and one receiving a simultaneous behavior tailoring condition (simultaneous condition) [46]. The only difference between the conditions was that in the sequential condition, respondents were invited to focus their attention first on a single behavior for which they failed to meet the Dutch national recommendations, whereas the simultaneous condition addressed all behaviors for which they failed to meet the Dutch recommendations at once. A detailed description of the study protocol has been published elsewhere [46].

### Participants and Procedure

**Figure 1:**
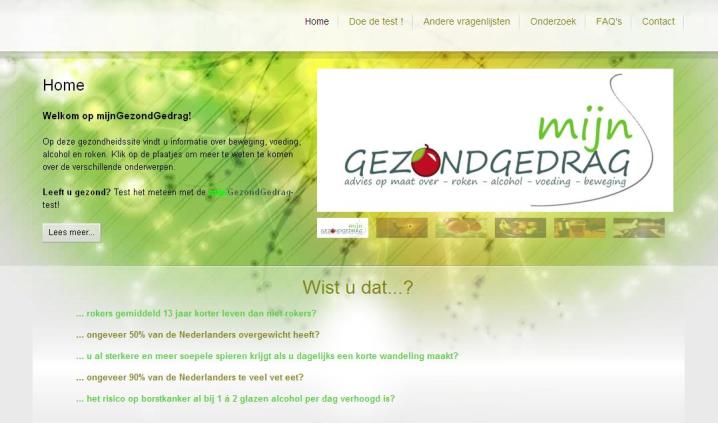
Screenshot of the homepage of the intervention program.

In the autumn of 2009, several Dutch Regional Health Authorities in the provinces of North-Brabant and Zeeland conducted an *Adult Health Monitor* study among adults (19-64 years) living in these provinces. This web-based questionnaire included demographics, aspects of general health and health-related topics. It also included questions regarding the respondents’ lifestyle behaviors (physical activity, fruit and vegetable consumption, alcohol intake, and smoking). Completion took an average of 36 minutes (SD = 15.8). At the end of the questionnaire, respondents received information about the tailored program. When interested in this program, they could fill in their e-mail address. The eligibility criteria were participation in the *Adult Health Monitor* study, a valid e-mail address, and computer / Internet literacy. Approximately 3 weeks after completing the monitor questionnaire, participants interested in receiving tailored feedback received an e-mail enabling them to log on to the computer tailored program (see Figure 1). After approximately one month, people who did not respond to this e-mail received a reminder e-mail.

### Intervention

The aim of the intervention was to stimulate participants to improve their lifestyle, focusing on 5 health behaviors. Based on the respondents’ answers to the different questions, an expert system selected the appropriate feedback messages from a large database and presented these directly on the respondent’s computer screen [46]. The I-Change model [47] was used as a theoretical framework for the questionnaires and the tailored advice.

**Figure 2:**
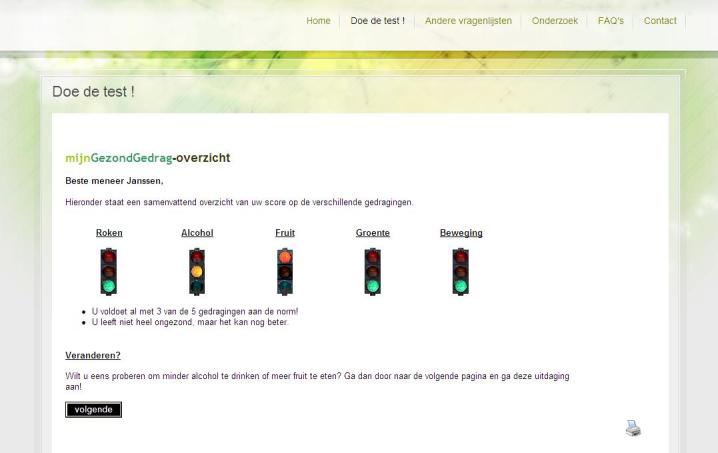
Screenshot of the health risk appraisal.

The first part of the feedback consisted of a health risk appraisal. Based on their answers on the *Adult Health Monitor* questionnaire, respondents received feedback concerning their lifestyle and information about whether they were meeting the public health guidelines defined for the 5 health behaviors, namely being moderately physically active for 30 minutes on at least five days a week; eating 200 g of vegetables per day; eating 2 pieces of fruit per day; not drinking more than 1 (women) or 2 (men) glasses of alcohol a day; and not smoking. In addition to more detailed information about the guidelines and the specific health behavior, respondents’ scores were depicted graphically in the form of a traffic light (indicating whether they met, almost met, or did not meet the guideline) as well a bar chart comparing the respondents’ behavior with the guideline for this behavior. At the end of the health risk appraisal, respondents received an overview illustrating their lifestyle behavior status (see Figure 2).

**Figure 3:**
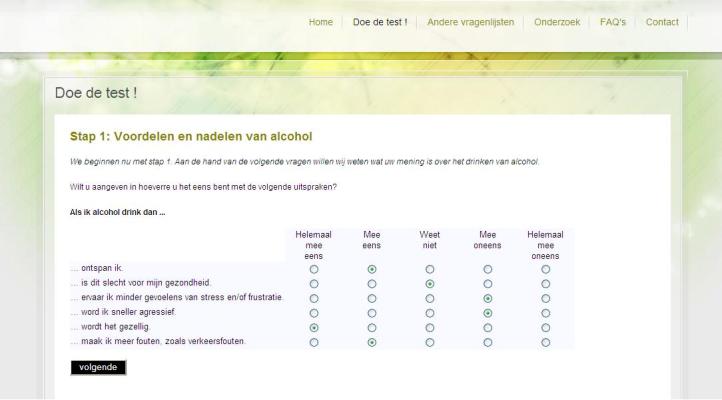
Screenshot of items regarding the pros and cons of alcohol intake.

**Figure 4:**
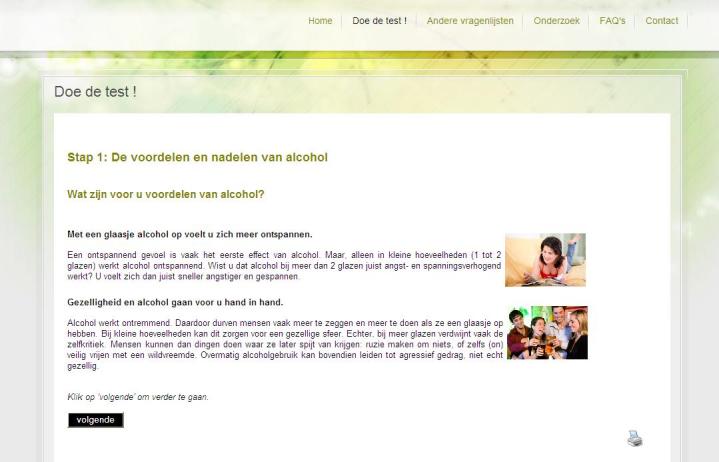
Screenshot of a personal advice regarding the pros of alcohol intake.

Afterwards, in the second part of the program, personal advice was provided, based on additional questions about psychosocial determinants (ie, attitude, social influence, preparatory action plans, self-efficacy, and coping plans; see Figures 3 and 4), on one or more lifestyle behaviors, depending on the tailoring condition.

#### Sequential Condition

After receiving the health risk appraisal, individuals in the sequential condition were invited to choose one of the health behaviors for which they were currently failing to meet the guideline. Respondents were encouraged to select the behavior that they were most motivated to change. This was followed by a progressive scheme consisting of 4 steps, in which respondents received personal advice based on various psychosocial constructs: (1) attitude, (2) social influence, (3) preparatory plans, and (4) self-efficacy and coping plans regarding the lifestyle behavior that they had chosen. Personal advice was given after the questions about each psychosocial construct (ie, attitude questions were followed by personal feedback about these items).

#### Simultaneous Condition

After receiving the health risk appraisal, participants in the simultaneous condition received feedback on all behaviors for which they failed to adhere to the public health guidelines in a predefined order. At random, half of the respondents started with the modules addressing preventive health behaviors (ie, (1) physical activity, (2) vegetable consumption, (3) fruit consumption) and ended with the modules addressing addiction behaviors (ie, (4) alcohol intake, (5) smoking), whereas the other half passed through the modules in reversed order. Respondents were presented with additional questions concerning psychosocial constructs, as well as personal advice on all behaviors for which they failed to adhere to the lifestyle recommendations. The 4-step progressive scheme ((1) attitude, (2) social influence, (3) preparatory plans, and (4) self-efficacy and coping plans) was used for all relevant lifestyle behaviors. Again, questions and personal advice were presented alternately.

### Measures

#### Demographic Information

The following demographic variables were assessed: age, gender, educational level (no education, primary, or lower vocational school (low); secondary vocational school or high school (medium); or higher professional education or university (high)), income, current job status, marital status, number of persons in the household, and country of origin.

#### Health Status

Quality of life was assessed using the SF-12 Health Survey [48,49]. Symptoms of depression and anxiety were assessed by the Kessler Psychological Distress Scale (K10) [50]. Body mass index (BMI) was estimated from questions about height and weight.

#### Lifestyle Behaviors

Five lifestyle behaviors were assessed using validated questionnaires: (1) physical activity, (2) fruit consumption, (3) vegetable consumption, (4) alcohol intake, and (5) smoking.

Physical activity was measured by the Short QUestionnaire to ASsess Health-enhancing physical activity (SQUASH) [51], and guideline adherence was assessed using procedures developed by Ainsworth et al. [52].

Fruit consumption was measured using a 4-item Food Frequency Questionnaire (FFQ) assessing weekly fruit and fruit juice intake [53].

Vegetable consumption was measured using a 4-item FFQ assessing the weekly consumption of boiled or baked vegetables, as well as salads or raw vegetables [53].

Alcohol intake was measured by the 5-item Dutch Quantity-Frequency-Variability (QFV) questionnaire [54].

Smoking was assessed by asking participants if they smoked, what they smoked (cigarettes, cigars, or pipe tobacco), and how much they smoked per day (cigarettes) or per week (cigars or pipe tobacco).

#### Psychosocial Determinants

The following description of the psychosocial determinants that were assessed is presented here to provide an overview of the program; the data on these items were not included in the analysis. Based on earlier studies [15], various psychosocial factors were assessed for the five different lifestyle behaviors: *attitude* (6 items, such as “Eating 2 pieces of fruit every day is good for my health” – totally disagree to totally agree); *social influence* (3 items, such as “How many people in your direct environment smoke?” – nobody to everybody); *self-efficacy* (6 items, such as “I am able to eat sufficient vegetables when I have other delicious food at home” – no, definitely not to yes, definitely); *preparatory plans* (3 items, such as “I intend to allow time for physical activity” – no, definitely not to yes, definitely); and *coping plans* (6 items, such as “I have made a plan to drink no more than 2 glasses of alcohol when I feel stressed or nervous” – totally disagree to totally agree).

#### Program Use

We counted the time respondents spent on the website during their first visit (ie, from logging in to the program until logging out or closing the website). Furthermore, we assessed the number of respondents who started with the first module and the number of respondents who filled out the program completely.

### Statistical Analyses

The data was analyzed using SPSS software, version 17.0. Descriptive statistics were used to describe the characteristics of the study sample and to calculate the dropout rates for the 2 tailoring conditions. In the sequential condition, a completer was defined as someone who filled in one module from start to finish (ie, including the final question) since the aim of the first visit was that respondents of this condition complete one module relating to a lifestyle behavior for which they failed to adhere to the guideline. In the simultaneous condition, a completer was defined as someone who completed all modules relating to the lifestyle behaviors for which they failed to adhere to the guidelines. The groups (ie, completers versus non-completers) were compared in terms of their demographics and lifestyle behaviors by means of Chi-square tests for discrete variables and independent-samples *t* tests for continuous variables. In addition, effect sizes (ES) were calculated based on means (Cohen’s *d*) and percentages (categorical variables). Effect sizes below 0.30 are considered small, while those between 0.30 and 0.80 are considered medium, and those larger than 0.80 are considered large [55]. Chi-square tests as well as effect size calculations were also used to explore differences between the tailoring conditions in terms of their completion rates, based on the number of guidelines that respondents failed to meet. Logistic regression analyses, using the Enter method, were used to identify predictors of program completion (demographics, health status, lifestyle behaviors and condition) within the entire sample. To identify interaction effects of tailoring condition and possible predictors, interaction terms were added to the regression equation. In the case of a significant interaction, logistic regression analyses were done separately for the two tailoring conditions to identify the predictors (demographics, health status, lifestyle behaviors).

## Results

### Participants’ Characteristics

A total of 3473 individuals participated in the present study. The mean age of the participants was 44 years. Slightly more men than women took part. With regard to the participants’ lifestyle, 17.4% (n = 608) failed to meet the physical activity guidelines, 67.4% (n = 2323) did not eat enough vegetables, 54.6% (n = 1873) did not eat enough fruit, 28.2% (n = 978) drank too much alcohol, and 19.0% (n = 660) reported that they smoked. Almost two-thirds did not adhere to two or more health behavior guidelines (n = 2106; 61.7%). The characteristics of the total sample are listed in Table 1.

**Table 1:** Demographics, health status and lifestyle of the study sample (N = 3473)

Variable		Total group
**Age**, n = 3473		43.61 (19-64; SD = 12.60)
**Gender**, n = 3473		
	Male	1849 (53.2%)
	Female	1624 (46.8%)
**Education**, n = 3458		
	Low	367 (10.6%)
	Medium	1607 (46.5%)
	High	1483 (42.9%)
**Income per month**, n = 3468		
	< € 1000	226 (6.5%)
	€ 1001 - € 1350	228 (6.6%)
	€ 1351 - € 1750	373 (10.8%)
	€ 1750 - € 3050	1177 (33.9%)
	> € 3051	976 (28.1%)
	“I don’t want to say”	488 (14.1%)
**Employment situation**, n = 3467		
	Employed	2655 (76.6%)
	Studying	229 (6.6%)
	Homemaker	176 (5.1%)
	Not currently in employment	407 (11.7%)
**Marital status**, n = 3457		
	Married	2092 (60.5%)
	Living together	528 (15.3%)
	Unmarried	639 (18.5%)
	Divorced	170 (4.9%)
	Widowed	28 (0.8%)
	# persons in household n = 3473	2.91 (1-20; SD = 1.42)
**Native country**, n = 3471		
	The Netherlands	3300 (95.1%)
	Other	171 (4.9%)
**BMI**, n = 3445		25.17 (15.03-58.11; SD = 3.96)
**Quality of Life**, n = 3452		40.11 (16-48; SD = 5.15)
**K10 (psychological distress)**, n = 3461		44.78 (12-50; SD = 5.70)
**Number of guidelines complied with**, n = 3411		
	0	25 (0.7%)
	1	226 (6.6%)
	2	681 (20.0%)
	3	1174 (34.4%)
	4	947 (27.8%)
	5	358 (10.5%)

**Physical activity**, n = 3473		
	Compliance	2865 (82.5%)
	Non-compliance	608 (17.4%)
**Vegetable consumption**, n = 3446		
	Compliance	1123 (32.6%)
	Non-compliance	2323 (67.4%)
**Fruit consumption**, n = 3433		
	Compliance	1560 (45.4%)
	Non-compliance	1873 (54.6%)
**Alcohol intake**, n = 3473		
	Compliance	2495 (71.8%)
	Non-compliance	978 (28.2%)
**Smoking**, n = 3473		
	Compliance	2813 (81.0%)
	Non-compliance	660 (19.0%)

### Completion and Dropout

**Figure 5:**
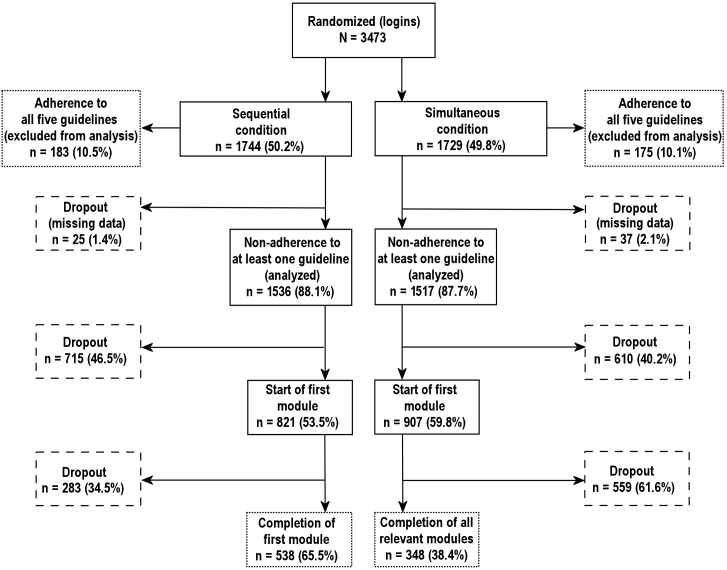
Attrition diagram.

As shown in Figure 5, the 3473 people who logged on to the program were evenly randomized to the 2 tailoring conditions. A total of 358 respondents (10.3%) adhered to all 5 health guidelines. These people were not included in our further analyses, as no specific completion moment could be defined for this group. Of the remaining 3115 respondents, 1325 (42.5%) logged out immediately after receiving the health risk appraisal, and 62 (2.0%) even before receiving the health risk appraisal: in the sequential condition, 53.5% (n = 821) started one lifestyle module, while in the simultaneous condition, 59.8% (n = 907) started at least the first lifestyle module (χ^2^_1_ = 12.48; *P* < .001). Of the 821 starters in the sequential condition, 65.5% (n = 538) completed the module, while of the 907 starters in the simultaneous condition, 38.4% (n = 348) completed the whole program (χ^2^_1_ = 127.25; *P* < .001).

On average, respondents in the sequential condition spent 10 minutes and 8 seconds on the web-based tailored program, while respondents in the simultaneous condition spent an average of 9 minutes and 47 seconds. In the sequential condition, respondents completed the program on average within 18 minutes and 10 seconds, while non-completers spent an average of 6 minutes and 20 seconds on the program. In the simultaneous condition, respondents completed the program within 20 minutes and 52 seconds, while non-completers left the program on average after 6 minutes and 16 seconds.

#### The Influence of Guideline Adherence Level on Dropout

**Figure 6:**
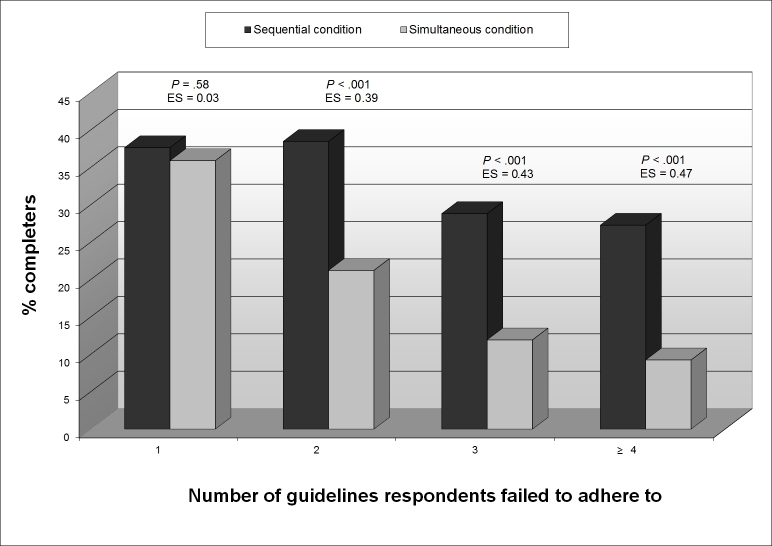
Number of guidelines respondents failed to adhere to against the number of completers in the sequential condition (n = 1536) and the simultaneous condition (n = 1517).

The completion rate generally decreased as the number of guidelines that the respondents failed to meet increased (see Figure 6). However, this decline of the completion rates differed between the two conditions. Respondents in the simultaneous condition who failed to adhere to 2 or more guidelines were more likely to leave the site prematurely than those in the sequential condition who failed to adhere to the same number of guidelines.

### Differences between Completers and Non-Completers

The 2 tailoring groups did not differ in terms of their demographics, health status or lifestyle behaviors, indicating that randomization had been successful. A comparison of respondents who filled in the entire program (ie, completers) with respondents who prematurely left the site (ie, non-completers) showed that the two groups differed on all variables, except for income, native country, K10 and alcohol intake (see Table 2). Medium effect sizes regarding these differences concerned age and the number of guidelines respondents adhered to. Completers were older than non-completers were and completers adhered to more health guidelines than non-completers did.

**Table 2:** Differences in demographics, health status and lifestyle between non-completers and completers (N = 3053)

Variable			Non-completer n = 2167	Completers n = 886	*T (P)*	*χ^2^ (P)*	*ES*
**Age**			41.85 (SD = 12.64)	47.21 (SD = 11.96)	11.07 (*P* < .001)		0.43
**Gender**							
	Male		1202 (55.5%)	450 (50.8%)			
	Female		965 (44.5%)	435 (49.2%)		6.58 (*P* = .037)	0.09
**Education**								
	Low		212 (9.8%)	120 (13.6%)			
	Medium		1007 (46.7%)	424 (48.0%)			
	High		938 (43.5%)	339 (38.4%)		13.35 (*P* = .010)	0.13
**Income per month ^a^**							
	< € 1750		504 (23.3%)	221 (25.0%)			
	€ 1751 - € 3050		1040 (48.0%)	437 (49.5%)			
	> € 3051		621 (28.7%)	225 (25.5%)		4.46 (*P* = .347)	0.08
**Employment situation**							
	Job		1852 (85.6%)	686 (77.7%)			
	No job		311 (14.4%)	197 (22.3%)		33.34 (*P* < .001)	0.21
**Relationship status**							
	Single		557 (25.8%)	191 (21.8%)			
	In relationship		1602 (74.2%)	687 (78.2%)		8.56 (*P* = .014)	0.11
**# persons in household**			2.99 (SD = 1.45)	2.74 (SD = 1.25)	-4.86 (*P* < .001)		0.18
**Native country**							
	The Netherlands		2075 (95.8%)	838 (94.7%)			
	Other		91 (4.2%)	46 (5.3%)		1.52 (*P* = .468)	0.05
**BMI**			25.00 (SD = 3.93)	25.54 (SD = 3.84)	3.47 (*P* = .001)		0.14
**Quality of Life**			40.19 (SD = 5.06)	39.39 (SD = 5.64)	-3.64 (*P* < .001)		0.15
**K10**			44.71 (SD = 5.75)	44.41 (SD = 6.12)	-1.27 (*P* = .203)		0.05
**Adherence to guidelines**			2.83 (SD = .96)	3.12 (SD = .88)	8.22 (*P* < .001)		0.31
	**Physical activity**						
		Compliance	1698 (78.4%)	761 (86.0%)			
		Non-compliance	469 (21.6%)	124 (14.0%)		23.62 (*P* < .001)	0.18
	**Vegetable consumption**						
		Compliance	501 (23.1%)	253 (28.6%)			
		Non-compliance	1666 (76.9%)	632 (71.4%)		10.43 (*P* = .005)	0.12
	**Fruit consumption**						
		Compliance	801 (37.0%)	388 (43.8%)			
		Non-compliance	1366 (63.0%)	497 (56.2%)		13.14 (*P* = .001)	0.13
	**Alcohol intake**						
		Compliance	1459 (67.3%)	632 (71.4%)			
		Non-compliance	708 (32.7%)	253 (28.6%)		5.32 (*P* = .070)	0.08

	**Smoking**						
		Compliance	1671 (77.1%)	731 (82.5%)			
		Non-compliance	496 (22.9%)	154 (17.4%)		11.56 (*P* = .003)	0.12
**Condition**							
	Sequential		998 (46.1%)	537 (60.7%)			
	Simultaneous		1169 (53.9%)	348 (39.3%)		54.74 (*P* < .001)	0.27

^a^ Note: Respondents who did not want to report their income were classified in the category “€ 1751 - € 3050”

### Predictors of Program Completion

We performed a logistic regression analysis to identify predictors of program completion. After the various interaction terms had been added, the interaction term ‘tailoring condition*non-adherence to guidelines’ emerged as statistically significant (B = -.620; *P* < .001) indicating that the effect of the number of guidelines respondents failed to adhere to on their completion status depended on the tailoring condition. Hence, separate analyses were performed for the 2 tailoring conditions. The results are presented in Table 3 (sequential condition) and Table 4 (simultaneous condition).

In model 1 of both conditions, the factors significantly associated with non-completion were a lower age and being male. In the simultaneous condition, Dutch nationality was also significantly associated with dropout. In model 2, the effect of age remained significant in both conditions. In the sequential condition, being male continued to make a significant contribution, whereas in the simultaneous condition, the gender and native country variables became non-significant. In both conditions, discontinuation of the program was predicted by the number of guidelines respondents failed to adhere to (in addition to a younger age). This means that people with a less healthy lifestyle were more likely to drop out than those with a healthier lifestyle. The second model of the sequential condition explained 8.2% of the total variance for program completion, whereas the second model of the simultaneous condition explained 15.1% of the total variance.

**Table 3:** Results of logistic regression analyses (Enter method) among the sequential condition on demographics and health status (model 1) and number of guidelines respondents failed to adhere to (model 2), with completion status (non-completers = 0; completers = 1) as dependent variable (N = 1496)

			Model 1				Model 2		
Variable			OR	*P*	CI		OR	*P*	CI
***Demographics***									
	**Age**		1.04	< .001	1.02-1.05		1.04	< .001	1.02-1.05
	**Gender**								
		Male (ref.)	1.00				1.00		
		Female	1.30	.02	1.04-1.63		1.27	.04	1.01-1.59
	** Education**								
		Low	1.42	.99	0.98-2.05		1.03	.90	0.69-1.53
		Medium	1.13	.36	0.86-1.50		1.14	.30	0.89-1.45
		High (ref.)	1.00				1.00		
	**Income**^a^**per month**								
		< € 1750	1.42	.06	0.98-2.05		1.42	.07	0.98-2.05
		€ 1751 - € 3050	1.13	.38	0.86-1.50		1.13	.40	0.84-1.49
		> € 3051 (ref.)	1.00				1.00		
	**Employment situation**								
		Job (ref.)	1.00				1.00		
		No job	1.01	.96	0.74-1.38		1.01	.97	0.73-1.38
	**Relationship status**								
		In relationship (ref.)	1.00				1.00		
		Single	0.94	.71	0.68-1.30		0.97	.83	0.70-1.33
	**# persons in household**		0.94	.18	0.87-1.03		0.94	.16	0.87-1.02
	**Native country**								
		The Netherlands (ref.)	1.00				1.00		
		Other	0.99	.93	0.57-1.66		0.99	.96	0.58-1.68
***Health status***									
		**BMI**	1.01	.57	0.98-1.04		1.01	.62	0.98-1.04
		**Quality of Life**	0.97	.07	0.94-1.00		0.97	.05	0.94-1.00
		**K10**	1.00	.75	0.98-1.03		1.00	.78	0.98-1.04
***Non-adherence to guidelines***									
	**Number of guidelines**						.86	.01	0.76-0.97
		Nagelkerke’s R^2^			.076				.082

^a^ Note: Respondents who did not want to report their income were classified in the category “€ 1751 - € 3050”

**Table 4:** Results of logistic regression analyses (Enter method) among the simultaneous condition on demographics and health status (model 1) and number of guidelines respondents failed to adhere to (model 2), with completion status (non-completers = 0; completers = 1) as dependent variable (N = 1473)

			Model 1				Model 2		
Variable			OR	*P*	CI		OR	*P*	CI
***Demographics***									
	**Age**		1.04	< .001	1.02-1.05		1.04	< .001	1.02-1.05
	**Gender**								
		Male (ref.)	1.00				1.00		
		Female	1.35	.03	1.04-1.74		1.13	.36	0.87-1.48
	**Education**								
		Low	1.30	.22	0.86-1.95		1.41	.11	0.92-2.16
		Medium	1.02	.88	0.77-1.35		1.09	.55	0.82-1.46
		High (ref.)	1.00				1.00		
	**Income**^a^**per month**								
		< € 1750	0.91	.65	0.60-1.38		0.87	.51	0.56-1.33
		€ 1751 - € 3050	0.89	.44	0.65-1.21		0.90	.51	0.65-1.24
		> € 3051 (ref.)	1.00				1.00		
	**Employment situation**								
		Job (ref.)	1.00				1.00		
		No job	1.18	.35	0.84-1.66		1.11	.56	0.78-1.58
	**Relationship status**								
		In relationship (ref.)	1.00				1.00		
		Single	1.10	.63	0.76-1.59		1.12	.55	0.77-1.65
		**# persons in household**	0.92	.11	0.82-1.02		0.90	.06	0.81-1.01
	**Native country**								
		The Netherlands (ref.)	1.00				1.00		
		Other	1.78	.04	1.03-3.08		1.54	.14	0.87-2.70
***Health status***									
	**BMI**		1.00	.79	0.96-1.03		1.00	.82	0.96-1.03
		**Quality of Life**	0.98	.35	0.95-1.02		0.98	.18	0.94-1.01
		**K10**	1.00	.82	0.97-1.04		1.00	.87	0.96-1.03
***Non-adherence to guidelines***									
	**Number of guidelines**						0.49	< .001	0.42-0.58
		Nagelkerke’s R^2^			.073				.151

^a^ Note: Respondents who did not want to report their income were classified in the category “€ 1751 - € 3050”

## Discussion

In view of the high number of people with an unhealthy lifestyle, there is a widely recognized need for interventions to change multiple behaviors. However, the best strategy to deliver such web-based interventions remains unclear. Addressing multiple health behaviors in one intervention leads to more extensive programs, which require more time and effort from the respondents [eg, 31]. We compared dropout rates of a sequential and a simultaneous version of a computer-tailored intervention regarding physical activity, fruit consumption, vegetable consumption, alcohol intake, and smoking, and investigated the predictive value of personal characteristics and lifestyle behaviors on completion and dropout rates for the 2 strategies.

Our first finding was that there were more non-completers in the simultaneous intervention than in the sequential intervention. The most important factor explaining the difference in dropout rate between these two conditions may be the difference in the length of the questionnaires and the computer-tailored advice that respondents received after the initial health risk appraisal. For example, if a respondent failed to adhere to 2 guidelines, the sequential intervention consisted of approximately 25 questions (average 10 minutes completion time), whereas the simultaneous intervention in such cases consisted of 50 questions, with an average completion time of 20 minutes. The advice also became twice as long, since the respondent had to fill in 2 modules in this case. Earlier research has also shown that the length of the program may be a primary reason to leave a website prematurely [22]. Another possible reason may be information overload [43]. Since each psychosocial construct is measured and tailored for each relevant behavior, this integrative approach is very demanding. An additional explanation that may need further research could be that in the simultaneous intervention, the 5 lifestyle modules had a predefined order, so respondents in this condition could not select the module they preferred to fill in first. Respondents in the sequential condition may have perceived more freedom of choice, since they could choose the lifestyle behavior about which they wanted to receive personal feedback. This hypothesis could be tested in follow-up studies, including qualitative interviews. 

Although the dropout rate was higher in the simultaneous intervention than in the sequential intervention, our findings revealed a high rate of non-completion in both types of intervention. One possible reason might be the recruitment strategy used. Completing the health risk appraisal took approximately 5 minutes in both conditions. The health risk appraisal was based on the *Adult Health Monitor* questionnaire that the respondents in our study had filled in at an earlier point in time. These respondents may not have wanted to make the effort of filling in a long questionnaire again. Hence, a considerable number of interested respondents of the potential target group may already have decided not to participate in the program after receiving the health risk appraisal. A second possible reason for the high dropout rate might be the study sample. Our study sample consisted of people from the general population, who were primarily invited simply to fill in a health-related questionnaire. Our study might have shown different results in terms of dropout rates in a group that would have been included based on their motivation to learn something about their lifestyle and/or to change lifestyle behaviors. This means that lack of motivation to change lifestyle may have been a reason for dropping out in both conditions [56,57]. Additionally, technical problems [58,59], e.g. disruption of the Internet connection or errors on the website, as well as problems navigating through the website, could have played a role – as was suggested by several e-mails received from respondents.

In terms of personal characteristics that were predictive of completion or non-completion of the program, significant influences were found of age and gender. Older people and women were more likely to complete the program, which is in line with earlier findings [25]. Furthermore, an unhealthy lifestyle was associated with higher dropout rates in both conditions. Earlier studies reported lower adherence to public health guidelines (ie, an unhealthier lifestyle) among people with a low socioeconomic status compared to people with a higher socioeconomic status [60,61], which means that this (high-risk) group in particular should be a target group for health promotion efforts. We found no difference in educational level, income, or employment status between respondents who dropped out at their first visit and those who did not. This is a relevant and promising finding, as it suggests that this tailored program is equally accepted and appreciated by both groups.

Our findings – with dropout rates being higher in the simultaneous condition than in the sequential condition – suggest that a sequential tailoring strategy might be able to reach the largest group of participants. However, since approximately 60-70% of the population fails to adhere to multiple public health guidelines, people may need information about more than one lifestyle behavior. The sequential strategy used in our intervention may therefore be insufficient to meet the needs of a large part of the population, especially those of people who are interested in several health behaviors and who are motivated to change multiple lifestyle behaviors. In our sequential intervention, respondents received the health risk appraisal, including information about the 5 health behaviors. Yet respondents were limited to one single module in the second part of the program at their first visit. In the long term, this approach can be regarded as a multiple behavior change intervention using a sequential strategy, but in the short term, detailed information is made available about one behavior only. Since the dropout rate at the very first visit was high, future research should first concentrate on prolonged use (ie, continuing the intervention for a substantial period of time) and possible information overload. To date, it seems to be a challenge to hold respondents’ attention in online interventions. Since the dropout rate even in the sequential condition is rather high, the number of psychosocial constructs as well as the tailored texts could be shortened, spread over time or delivered in different forms. Including more interactive elements, such as videos or games, may improve the attractiveness of eHealth programs, which in turn may result in longer visits [62-64]. Stimulating re-visits, which are necessary in our sequential approach, poses a second challenge for future research.

The simultaneous tailoring strategy has advantages as well, insofar as people may receive tailored feedback on more than one lifestyle behavior at once. However, it may be better not to offer the modules in a predefined order. A study by Brouwer et al. [25] shows that when people have a choice to select more than one behavior, they make use of this option and choose different behavior modules.

Another option to explore is a mixture of both tailoring strategies, called preference-based tailoring [39,65] in which respondents can select the behavior modules which they want to fill in (not limited to only one module). This may make respondents perceive a higher level of autonomy [66,67] since they would not have to limit themselves to one single behavior at first, and could receive as much information as they wish.

### Strengths, Limitations, and Recommendations

To our knowledge, this is the first study to compare sequential and simultaneous interventions addressing the 5 lifestyle behaviors of physical activity, fruit consumption, vegetable consumption, alcohol intake, and smoking, in terms of dropout rates. The study has yielded new information about predictors of completion of the 2 intervention types.

The findings of this study should be interpreted keeping several limitations in mind. Our findings were based on self-reports, which could have led to recall bias (e.g., the high proportion of people who reported to meet the physical activity guideline may represent an overestimation of their actual level of physical activity); and the amounts of variance explained by our regression models were relatively low, indicating that other factors might play a role in determining program completion. Future research is necessary to identify additional relevant factors, for example, motivation to change, available time, interest in the topic, program evaluation (in terms of, eg, user-friendliness and attractiveness), and expectations from the program.

The present study provides initial evidence for higher attrition rates in the simultaneous intervention strategy. Although this is likely to result in lower effectiveness of this intervention, future studies need to address the relative efficacy and effectiveness of simultaneous versus sequential tailoring. Hence, re-visiting rates for the two types of interventions should be compared, and the differences in effectiveness in terms of successful behavior change should be tested. It is imaginable that despite the higher dropout in the simultaneous condition, more respondents in this condition received all relevant information compared to those in the single/sequential condition who possibly only read information about the most preferred behavior module and/or never return to the intervention program. More research remains to be done to study in which condition more modules are opened and/or completed by the respondents during the duration of the project.

### Conclusions

Our findings indicate a high rate of non-completion in both types of intervention, with more incompletes in the simultaneous intervention and among respondents with unhealthier lifestyles. In both conditions, discontinuation of the program was related to a younger age of the respondent, and in the sequential condition, being male was also associated with non-completion of the program. The results of this study suggest opportunities for optimizing online tailored lifestyle interventions: such programs should be tailored to all individual users; their efficiency should be improved; their attractiveness should be enhanced by integrating interactive elements; and their content and length or duration should be balanced.

## Multimedia Appendix 1

CONSORT EHEALTH checklist V1.6 [68].
