# Supplementary material for: Program Completion of a Web-Based Tailored Lifestyle Intervention for Adults: Differences between a Sequential and a Simultaneous Approach
Source: J Med Internet Res. 2012 Mar 8;14(2):e26. doi: 10.2196/jmir.1968 (PMC3376514; doi:10.2196/jmir.1968)
Supplement: Supplementary file 1 [file jmir_v14i2e26_app1.pdf]

CONSORT-EHEALTH Checklist V1.6 Report

Manuscript Number 1968

Date completed 8/25/2011 14:50:29

by Daniela Schulz

Program completion of a web-based tailored lifestyle intervention for adults: differences between a sequential and a simultaneous approach

TITLE

1a-i) Identify the mode of delivery in the title

YES:
"Program completion of a web-based tailored lifestyle intervention [...]"

1a-ii) Non-web-based components or important co-interventions in title

n/a

1a-iii) Primary condition or target group in the title

YES:
"[...] tailored lifestyle intervention for adults [...]"

ABSTRACT

1b-i) Key features/functionalities/components of the intervention and comparator in the METHODS section of the ABSTRACT

YES:
"[...] demographics, health status, physical activity, vegetable consumption, fruit consumption, alcohol intake and smoking were self-assessed through questionnaires [...] a health risk appraisal was offered [...] psychosocial determinants of the lifestyle behaviors were assessed and personal advice was provided, about one or more lifestyle behaviors."

1b-ii) Level of human involvement in the METHODS section of the ABSTRACT

1b-iii) Open vs. closed, web-based (self-assessment) vs. face-to-face assessments in the METHODS section of the ABSTRACT

"[...] self-assessed [...]"

1b-iv) RESULTS section in abstract must contain use data

"Our findings indicate a high non-completion rate for both types of intervention (71.0%; n = 2,167), with more incompletes in the simultaneous intervention (77.1%; n = 1,169) than in the sequential intervention (65.0%; n = 998). In both conditions, discontinuation was predicted by a lower age (sequential condition: OR = 1.04; P < .001; CI = 1.02-1.05; simultaneous condition: OR = 1.04; P < .001; CI = 1.02-1.05) and an unhealthy lifestyle (sequential condition: OR = .86; P = .01; OR = .76-.97; simultaneous condition: OR = .49; P < .001; CI = .42-.58). In the sequential intervention, being male (OR = 1.27; P = .04; CI = 1.01-1.59) also predicted drop-out."

1b-v) CONCLUSIONS/DISCUSSION in abstract for negative trials

"Possible reasons for the higher drop-out rate in our simultaneous intervention may be the amount of time required and information overload. Strategies to optimize program completion as well as continued use of computer-tailored interventions should be studied."

INTRODUCTION

2a-i) Problem and the type of system/solution

Our intervention program is intended as a stand-alone intervention for adults of the general population.

"Internet-based programs may have the potential to reach large numbers of people. However, various studies have pointed out that the actual use of web-based interventions may be limited [19-21] and that leaving an Internet intervention prematurely is common [e.g. 22-24]. [...] Hence, there is a need to identify factors associated with early discontinuation or continuation of participation in web-based programs promoting the adoption of healthy lifestyles. [...] To our knowledge, there is no literature about the difference in completion and drop-out rates between users of sequential and those of simultaneous behavior change interventions. [...] it is unknown how much information people can handle in multiple behavior change interventions."

2a-ii) Scientific background, rationale: What is known about the (type of) system

"Various tailoring strategies can be used to address multiple behaviors with computer-tailored interventions, such as a sequential or a simultaneous strategy. Using a simultenous strategy, different behaviors are intervened on concurrently; using a sequential strategy, one behavior is treated at a time. Few studies have investigated the effects on behavioral change of sequential versus simultaneous strategies to provide multiple health behavior change interventions, and these studies reported inconsistent findings [26-28]. According to Vandelanotte et al. [28], the sequential strategy may be more effective than the simultaneous strategy when participants can choose the behavior on which they would prefer to receive personal feedback first, and can start with this part of the intervention, instead of the lifestyle modules being presented in a predefined order [see also 29].
In any case, behavioral change will be more likely when someone completes the whole intervention program [30], as early drop-out is a hazard to the effectiveness of any intervention. Hence, when considering the use of a sequential or simultaneous approach for web-based computer-tailored interventions, it is important to study continuation rates. In both tyes of intervention, people receive only the modules about health behavior topics for which they are at risk in order to increase the relevance of the intervention [31]. When being at risk for at least two behaviors, people in the simultaneous intervention receive, and thus have to handle, more information at one point in time compared to a sequential intervention, in which the same amount of information is spread over time. Therefore, a simultaneous strategy, including a more complex program, is likely to require more time from the respondents and to increase the behavior change demands [31], especially when respondents fail to meet multiple guidelines. Hence, such a strategy may lead to higher drop-out rates than a sequential strategy, potentially because a simultaneous approach may lead to an overload of information [31-32]. Respondents may become overwhelmed by the amount of information [33] and may perceive ego depletion, leading to a reduced capacity to change [34]. Furthermore, tailoring multiple behaviors simultaneously could fail to address any single behavior in sufficient depth [3,31,35]. On the other hand, addressing various behaviors simultaneously may optimize the occurrence of synergistic effects [36-40]. [...] In a study by Brouwer et al. [25], respondents who completed the program were mostly women, middle-aged (40 to 50 years) and medium-educated, and had a healthier lifestyle."

METHODS

3a) CONSORT

"In this study, we investigated the level of completion of a web-based tailored intervention addressing five lifestyle behaviors (physical activity, fruit consumption, vegetable consumption, alcohol intake, and smoking), and tested potential information overload by comparing drop-out rates for two versions of the program, one offering a single behavior change module as part of a sequential program and one providing simultaneous tailored feedback on the different behaviors. In addition, this study investigated personal predictors of drop-out for the two versions of our computer-tailored program."

3b-i) Bug fixes, Downtimes, Content Changes

We sent one additional (not planned) reminder in order to increase the response.

4a-i) Computer / Internet literacy

"The eligibility criteria were: [...] computer / Internet literacy."

4a-ii) Open vs. closed, web-based vs. face-to-face assessments:

"This web-based questionnaire" [...] "At the end of the questionnaire, respondents received information about the tailored program. When interested in this program, they could fill in their e-mail address. [...] Approximately three weeks after completing the monitor questionnaire, participants interested in receiving tailored feedback received an e-mail enabling them to log on to the computer tailored program."

4a-iii) Information giving during recruitment

4b-i) Report if outcomes were (self-)assessed through online questionnaires

"Based on their answers on the Adult Health Monitor questionnaire, [...] Based on the respondents' answers to the different questions, [...] The following demographic variables were assessed [...]"

4b-ii) Report how institutional affiliations are displayed

n/a

5-i) Mention names, credential, affiliations of the developers, sponsors, and owners

5-ii) Describe the history/development process

We had executed a pilot study in another province in the Netherlands (in Limburg).

5-iii) Revisions and updating

n/a - no major changes

5-iv) Quality assurance methods

5-v) Ensure replicability by publishing the source code, and/or providing screenshots/screen-capture video, and/or providing flowcharts of the algorithms used

We included some screenshots in the manuscript.

5-vi) Digital preservation

We archived the intervention site (webcitation 63PfyjS9k).

5-vii) Access

Respondents of the Adult Health Monitor 2009 were allowed to take part in our study. Participation was free of charge and respondents are eligible to win prizes (see study protocol:
Schulz DN, Kremers SP, Van Osch LA, Schneider F, Van Adrichem MJ, De Vries H. Testing a Dutch web-based tailored lifestyle Program among adults: a study protocol. BMC Public Health 2011;11:108. PMID: 21324181)

"Approximately three weeks after completing the monitor questionnaire, participants interested in receiving tailored feedback received an e-mail enabling them to log on to the computer tailored program "

5-viii) Mode of delivery, features/functionalities/components of the intervention and comparator, and the theoretical framework

"The I-Change model was used as a theoretical framework for the questionnaires and the tailored advice. The first part of the feedback consisted of a health risk appraisal. Based on their answers on the Adult Health Monitor questionnaire, respondents received feedback concerning their lifestyle and information about whether they were meeting the public health guidelines defined for the five health behaviors, [...] In addition to more detailed information about the guidelines and the specific health behavior, respondents' scores were depicted graphically in the form of a traffic light (indicating whether they met, almost met or did not meet the guideline) as well a bar chart comparing the respondents' behavior with the guideline for this behavior. At the end of the health risk appraisal, respondents received an overview illustrating their lifestyle behavior status. Afterwards, the second part of the program started, in which personal advice was provided based on additional questions about psychosocial determinants (i.e., attitude, social influence, preparatory action plans, self-efficacy and coping plans; see Figures 3 and 4) on one or more lifestyle behaviors, depending on the tailoring condition.

Sequential condition
After receiving the health risk appraisal, individuals in the sequential condition were invited to choose one of the health behaviors for which they were currently failing to meet the guideline. Respondents were encouraged to select the behavior which they were most motivated to change. This was followed by a progressive scheme consisting of four steps, in which respondents received personal advice based on various psychosocial constructs: (1) attitude, (2) social influence, (3) preparatory plans and (4) self-efficacy and coping plans regarding the lifestyle behavior that they had chosen. Personal advice was given after the questions about each psychosocial construct (i.e., attitude questions were followed by personal feedback about these items).

Simultaneous condition
After receiving the health risk appraisal, participants in the simultaneous condition received feedback on all behaviors for which they failed to adhere to the public health guidelines in a predefined order. At random, half of the respondents started with the modules addressing preventive health behaviors (i.e., (1) physical activity, (2) vegetable consumption, (3) fruit consumption) and ended with the modules addressing addiction behaviors (i.e., (4) alcohol intake, (5) smoking), whereas the other half passed through the modules in reversed order. For those behaviors for which respondents failed to adhere to the lifestyle recommendations, they were presented with additional questions concerning psychosocial constructs as well as personal advice on all these behaviors. The four-step progressive scheme ((1) attitude, (2) social influence, (3) preparatory plans and (4) self-efficacy and coping plans) was used for all relevant lifestyle behaviors. Again, questions and personal advice were presented alternately."

We also added some example items and added screenshots of the website / items / advice.

5-ix) Describe use parameters

On the website, respondents could find information about the number of e-mails/invitations they will receive and about the duration of the study.

5-x) Clarify the level of human involvement

5-xi) Report any prompts/reminders used

"Approximately three weeks after completing the monitor questionnaire, participants interested in receiving tailored feedback received an e-mail enabling them to log on to the computer tailored program. After approximately one month, people who did not respond to this e-mail, received a reminder e-mail."

5-xii) Describe any co-interventions (incl. training/support)

n/a - Our intervention was a stand-alone intervention.

6a-i) Online questionnaires: describe if they were validated for online use and apply CHERRIES items to describe how the questionnaires were designed/deployed

6a-ii) Describe whether and how "use" (including intensity of use/dosage) was defined/measured/monitored

"Program use
We counted the time respondents spent on the website during their first visit (i.e. from logging in to the program until logging out or closing the website). Furthermore, we assessed the number of respondents who started with the first module and the number of respondents who filled out the program completely."

6a-iii) Describe whether, how, and when qualitative feedback from participants was obtained

We added a feedback questionnaire on the website which could be filled in at every moment. Moreover, we received some e-mails from respondents: "[...] technical problems, e.g. disruption of the Internet connection or errors on the website, as well as problems navigating through the website, could have played a role – as was suggested by several e-mails received from respondents."

7a-i) Describe whether and how expected attrition was taken into account when calculating the sample size

7b) CONSORT

n/a

8a) CONSORT

We used a syntax within the software-program in order to randomize the respondents among the different groups (called projects in the software-program).

8b) CONSORT

Randomization took place at the individual level; no blocking was used.

9) CONSORT

Also the control group got a small amount of personlized information in order to conceal allocation to the control group. However, this item is not that relevant since we focus on the two experimental groups only (in our manuscript).

10) CONSORT

Participants of the Adult Health Monitor were allowed to take part in our study. The computer-tailoring software, facilitated by the software-company, was used for the randomization process.

11a-i) Specify who was blinded, and who wasn't

Our respondents were blinded.

11a-ii) Discuss e.g., whether participants knew which intervention was the "intervention of interest" and which one was the "comparator"

11b) CONSORT

n/a

12a) CONSORT

"The data was analyzed using SPSS software, version 17.0. Descriptive statistics were used to describe the characteristics of the study sample and calculate the drop-out rates for the two tailoring conditions. In the sequential condition, a completer was defined as someone who filled in one module from start to finish (i.e. including the final question) since the aim of the first visit was that respondents of this condition completed one module relating to a lifestyle behavior for which they failed to adhere to the guideline. In the simultaneous condition, a completer was defined as someone who completed all modules relating to the lifestyle behaviors for which they failed to adhere to the guidelines. The groups (i.e., completers versus non-completers) were compared in terms of their demographics and lifestyle behaviors by means of Chi-square tests for discrete variables and independent-samples t-tests for continuous variables. In addition, effect sizes were calculated based on means (Cohen's d) and percentages (categorical variables). Effect sizes below .30 are considered small, while those between .30 and .80 are considered medium, and those larger than .80 are considered large [55]. Chi-square tests as well as effect size calculations were also used to explore differences between the tailoring conditions in terms of their completion rates, based on the number of guidelines respondents failed to meet. Logistic regression analyses, using the Enter method, were used to identify predictors of program completion (demographics, health status, lifestyle behaviors and condition) among the whole sample. To identify interaction effects of tailoring condition and possible predictors, interaction terms were added to the regression equation. In the case of a significant interaction, logistic regression analyses were done separately for the two tailoring conditions to identify the predictors (demographics, health status, lifestyle behaviors)."

12a-i) Imputation techniques to deal with attrition / missing values

The statistical method depends on the variable; missings on primary outcomes (health behavior guidelines) were left as missings; or missings were imputed to the median (e.g. income-variable).

12b) CONSORT

"Logistic regression analyses, using the Enter method, were used to identify predictors of program completion (demographics, health status, lifestyle behaviors and condition) among the whole sample. To identify interaction effects of tailoring condition and possible predictors, interaction terms were added to the regression equation. In the case of a significant interaction, logistic regression analyses were done separately for the two tailoring conditions to identify the predictors (demographics, health status, lifestyle behaviors)."

RESULTS

13a) CONSORT

We added an attrition diagram in which these results are shown.

13b) CONSORT

We added an attrition diagram in which these results are shown. We do not know the reasons for drop-outs.

13b-i) Attrition diagram

YES: we added an attrition diagram.

14a) CONSORT

We only had a look at our baseline measurement (2009).

14a-i) Indicate if critical "secular events" fell into the study period

14b) CONSORT

n/a

15) CONSORT

YES, see Table 1 and Table 2.

15-i) Report demographics associated with digital divide issues

YES, see Table 1 and Table 2.

16-i) Report multiple "denominators" and provide definitions

See Figure 5 (flow-chart)

16-ii) Primary analysis should be intent-to-treat

17a) CONSORT

See Table 2

17a-i) Presentation of process outcomes such as metrics of use and intensity of use

"On average, respondents in the sequential condition spent 10 minutes and 8 seconds on the web-based tailored program, while respondents in the simultaneous condition spent an average of 9 minutes and 47 seconds. In the sequential condition, respondents completed the program on average within 18 minutes and 10 seconds, while non-completers spent an average of 6 minutes and 20 seconds on the program. In the simultaneous condition, respondents completed the program within 20 minutes and 52 seconds, while non-completers left the program on average after 6 minutes and 16 seconds."

17b) CONSORT

See Table 2 and Figure 5.

18) CONSORT

"The influence of guideline adherence level on drop-out
The completion rate generally decreased as the number of guidelines that the respondents failed to meet increased (see Figure 6). However, this decline of the completion rates differed between the two conditions. Respondents in the simultaneous condition who failed to adhere to two or more guidelines were more likely to leave the site prematurely than those in the sequential condition who failed to adhere to the same number of guidelines.

Differences between completers and non-completers
The two tailoring groups did not differ in terms of their demographics, health status or lifestyle behaviors, indicating that randomization had been successful. A comparison of respondents who filled in the entire program (i.e., completers) with respondents who prematurely left the site (i.e., non-completers) showed that the two groups differed on all variables, except for income, native country, K10 and alcohol intake (see Table 2). Medium effect sizes regarding these differences concerned age and the number of guidelines respondents complied with. Completers were older than non-completers and completers adhered to more health guidelines than non-completers."

18-i) Subgroup analysis of comparing only users

19) CONSORT

No intended harms were reported.

19-i) Include privacy breaches, technical problems

It might be that technical problems occured (in all groups).

19-ii) Include qualitative feedback from participants or observations from staff/researchers

DISCUSSION

20-i) Typical limitations in ehealth trials

"The findings of this study should be interpreted keeping several limitations in mind. Our findings were based on self-reports, which could have led to recall bias (e.g., the high proportion of people who reported to meet the physical activity guideline may represent an overestimation of their actual level of physical activity); and the amounts of variance explained by our regression models were relatively low, indicating that other factors might play a role in determining program completion."

21-i) Generalizability to other populations

All respondents filled in the Adult Health Monitor (inclusion criterion) before taking part in our intervention program. We cannot ensure generalizability of our results to other populations.
"[...] the high proportion of people who reported to meet the physical activity guideline may represent an overestimation of their actual level of physical activity" - or it could be that most of our respondents already is physically active, which means that our group is not representative for the general population in the Netherlands.

21-ii) Discuss if there were elements in the RCT that would be different in a routine application setting

22-i) Restate study questions and summarize the answers suggested by the data, starting with primary outcomes and process outcomes (use)

"In view of the high number of people with an unhealthy lifestyle, there is a widely recognized need for interventions to change multiple behaviors, but the best strategy to deliver such web-based interventions still remains unclear. Addressing multiple health behaviors in one intervention leads to more extensive programs which require much time and effort of the respondents [e.g. 31]. We compared drop-out rates of a sequential and a simultaneous version of a computer-tailored intervention regarding physical activity, fruit consumption, vegetable consumption, alcohol intake and smoking, and investigated the predictive value of personal characteristics and lifestyle behaviors on completion and drop-out rates for the two strategies.
Our first finding was that there were more non-completers in the simultaneous intervention than in the sequential intervention. The most important factor explaining the difference in drop-out rate between these two conditions may be the difference in the length of the questionnaires and the computer-tailored advice which respondents received after the initial health risk appraisal. [...] Another possible reason may be information overload [43]. [...] Although the drop-out rate was higher in the simultaneous intervention than in the sequential intervention, our findings revealed a high rate of non-completion in both types of intervention. [...] In terms of personal characteristics that were predictive of completion or non-completion of the program, significant roles were played by age and gender. Older people and women were more likely to complete the program, which is in line with earlier findings [25]. Furthermore, an unhealthy lifestyle was associated with higher drop-out rates in both conditions."

22-ii) Highlight unanswered new questions, suggest future research

“An additional explanation that may need further research could be that in the simultaneous intervention, the five lifestyle modules had a predefined order, so respondents in this condition could not select the module they preferred to fill in first. Respondents in the sequential condition may have perceived more freedom of choice, since they could choose the lifestyle behavior about which they wanted to receive personal feedback. This hypothesis could be tested in follow-up studies, including qualitative interviews. [...] The present study provides initial evidence for higher attrition rates in the simultaneous intervention strategy. Although this is likely to result in lower effectiveness of this intervention, future studies need to address the relative efficacy and effectiveness of simultaneous versus sequential tailoring. Hence, re-visiting rates for the two types of interventions should be compared, and the differences in effectiveness in terms of successful behavior change should be tested. It is imaginable that despite the higher drop-out in the simultaneous condition, more respondents in this condition got to receive all relevant information compared to those in the single/sequential condition who possibly only read information about the most prefered behavior module and/or never return to the intervention program. More research remains to be done to study in which condition more modules are opened and/or completed by the respondents during the duration of the project. [...] Future research is necessary to identify additional relevant factors, for example motivation to change, available time, interest in the topic, program evaluation (in terms of, e.g., user-friendliness and attractiveness) and expectations from the program. [...] The results of this study suggest opportunities for optimizing online tailored lifestyle interventions: such programs should be tailored to all individual users; their efficiency should be improved; their attractiveness should be enhanced by integrating interactive elements; and their content and length or duration should be balanced."

Other information

23) CONSORT

Dutch Trial Register NTR2168.

24) CONSORT

Schulz DN, Kremers SP, Van Osch LA, Schneider F, Van Adrichem MJ, De Vries H. Testing a Dutch web-based tailored lifestyle Program among adults: a study protocol. BMC Public Health 2011;11:108. PMID: 21324181

25) CONSORT

"This study was funded by ZonMw, the Netherlands Organisation for Health Research and Development (grant number: 120610012). Intervention development and implementation took place at Maastricht University. Data collection and data analysis were done in collaboration with the Regional Health Authorities of the Dutch provinces of North-Brabant (GGD Brabant-Zuidoost; GGD Hart voor Brabant; and GGD West-Brabant) and Zeeland (GGD Zeeland)."

X26-i) Comment on ethics committee approval

x26-ii) Outline informed consent procedures

X26-iii) Safety and security procedures

X27-i) State the relation of the study team towards the system being evaluated
